# The quality characteristics of air-dried cured meat (Grison) processed from cattle, buffalo, and camel: a comparative study

**DOI:** 10.1038/s41598-026-54134-7

**Published:** 2026-05-27

**Authors:** Mostafa A. Gouda, Mai A. Mohamed, Mohamed M. T. Emara, Marwa R. S. Abdallah

**Affiliations:** Department of Food Hygiene and Control, Faculty of Veterinary Medicine, Cairo University, Giza, 12211 Egypt

**Keywords:** Non-traditional meat products, Air-dried meat, Different types of meat, Grison, Instrumental color, Residual nitrite, Salt concentration, Oxidation, Sensory quality, Biochemistry, Zoology

## Abstract

Dried meat products have recently gained popularity due to their prolonged shelf life, nutritional advantages, and characteristic sensory quality. The main objective of this study was to assess the quality of air-dried cured meat (Grison) produced from cattle, buffalo, and camel meat, with a special focus on the impact of the meat from different species of animal on the quality of the meat during the curing process and in the finished product. Fresh topside from each species was dry-cured under a controlled environment with evaluation of meat samples throughout the curing period, and additional tests were performed on the finished products. The proximate composition, pH value, lipid oxidation, salt concentration, residual nitrite content, instrumental color indices, shear force value, and sensory attributes were evaluated. The findings revealed many noteworthy differences in the air-dried cured meat produced with various types of meat during both the curing phase and in the end product. Camel meat and its corresponding Grison exhibited higher moisture, pH value, residual nitrite content, redness (a*), and chroma (C*) values, but lower protein, fat, TBARS, and hue angle (*h°*) values compared to cattle and buffalo counterparts. Cattle Grison showed higher fat content, lightness (L*), yellowness (b*), and (*h*°) values, but a lower ΔE value, whereas buffalo Grison exhibited a higher shear force value. Sensory assessment revealed that buffalo Grison exhibited the lowest juiciness and tenderness, while camel Grison showed the highest color and flavor scores. Overall, Grison made from camel meat was comparable to that of the traditional cattle one and superior in numerous quality features to that of buffalo; however, more optimization is needed to improve the quality of buffalo-based air-dried cured meat products, particularly tenderness.

## Introduction

Meat preservation is fundamental in addressing the global food shortages because fresh meat is highly perishable due to its high moisture and protein content, which provide an ideal medium for microbial proliferation^1^. Drying meat is a traditional preservation technique that has been practiced in various cultures worldwide^2^. Various dry-cured meat products are produced globally, with each variant characterized by both the raw meat materials and the processing techniques used^3^. One notable example is Viande des Grisons, also known as “Bunderfleisch,” which originated in the Alpine regions, particularly in the canton of Graubünden, Switzerland. The Grison is prepared from cattle hindquarter cuts such as topside or silverside, which undergo salting, seasoning with herbs and spices, followed by air-drying under controlled environmental conditions^4^. Although Grison is predominantly consumed in Switzerland, it is also exported to several European countries, as well as to Canada, the United States, and Japan, and has recently become available in Egypt.

Drying is the process of slowly removing water from meat under controlled sanitary conditions, which results in a shelf-stable lean product with intense red color, high protein, low fat content, firm texture, and a characteristic, powerful flavor. The air-drying of meat also preserves vitamins, minerals, and other useful ingredients and results in a product that does not require refrigeration or preservatives^5^. In recent years, several alternative drying technologies have been developed to avoid the limitations encountered during the conventional drying process and to obtain high-quality dried meat products. However, air drying remains the method of choice among the other drying techniques because the use of methods that depend on higher temperatures is claimed to diminish valuable nutrients, deteriorate the flavor, and adversely impact the texture of the final product^6^.

The characteristic quality attributes of air-dried cured meat develop during the processes of curing, drying, and ripening. The flavor of air-dried cured meat is due to the metabolites produced as a result of the action of both lipolytic and proteolytic enzymes on meat during the ripening process, while the substantial and steady moisture loss during the drying phase gives the meat its firm texture, especially on the surface^7^. The protein denaturation causes the majority of changes in meat during drying; in particular, the product becomes darker due to oxidation of myoglobin pigments and denaturation of heme proteins. The protein denaturation also causes shrinkage of muscle fibers, a decrease in water-holding capacity, and firmness of the meat texture^8^.

Traditionally, cattle meat was the principal raw material for the production of Grison. However, consumers’ desire for more sustainable and healthful protein sources, along with the continuous increase in cattle costs increased attention to other meat animals. Buffalo meat is generally leaner than cattle, with lower intramuscular fat and higher protein content^9^, while the camel meat typically contains higher moisture and protein levels than cattle, which generally exhibits higher fat content^10^. The physicochemical quality of buffalo meat has also been reported to be comparable to that of cattle. Neath et al.^11^ reported a slower post-mortem pH decline in buffalo meat than in cattle; while cattle reached the ultimate pH value within 24 h post-mortem, buffalo meat attained a similar pH value after approximately 48 h, which corresponded with higher WHC compared to cattle^12^. The camel meat has consistently been reported to exhibit a slower post-mortem pH decline and a higher ultimate pH value compared with cattle. This difference has been mainly attributed to the lower glycogen reserves and reduced glycolytic enzyme activity in camel muscles^13^. In line with its higher ultimate pH value, camel meat has been shown to exhibit a superior WHC^14^.

The meat color is another critical quality attribute that influences consumer purchasing decisions and perceptions of freshness. Buffalo meat exhibits a higher iron and myoglobin content than cattle^15^, which contributes to its higher redness (a*) value. In contrast, several studies have reported higher yellowness (b*) values in buffalo meat, which has been associated with a greater proportion of met-myoglobin and lower proportions of deoxy-myoglobin and oxy-myoglobin compared with cattle^16^. Moreover, camel meat exhibits distinct color characteristics governed by intrinsic factors such as myoglobin concentration, muscle fiber composition, and post-mortem pH behavior. In this regard, camel meat has been reported to show significantly lower (L*) values than cattle, while exhibiting higher (a*) and yellowness (b*) values, resulting in a darker appearance^14^. Despite this advantage, both camel and buffalo meat are recognized as being tougher than cattle, as indicated by their higher shear force due to variations in WHC, collagen content, and collagen solubility^17,18^.

Regardless of their comparable nutritional quality, buffalo and camel meat are of particular interest due to their unique physical properties, compositional, and functional attributes that are expected to influence drying kinetics, texture, color stability, and oxidative behavior in air-dried cured meat products, moreover, the presence of bioactive substances like riboflavin, niacin, glutathione oxidase, and catalase, in both buffalo and camel meat have recently drawn increasing attention as a promising alternative for processing the traditional air-dried meat products^19^.

Although numerous studies have been conducted to investigate the impact of various drying processes on the quality of air-dried meat products, comparative studies evaluating the effect of meat from different species of animals on the quality of air-dried meat products are noticeably scarce. Research has primarily focused on cattle^20^ and buffalo to some extent^21^. However, the utilization of camel meat in air-dried or dry-cured products remains underexplored. Furthermore, research on Grison processing and quality is rare. It is hypothesized that differences in raw meat characteristics among animal species will lead to significant variations in the quality characteristics of air-dried meat during the curing process and in the final product. Therefore, this study aims to produce one of the most valuable and well-known traditional air-dried meat, “Viande des Grisons”, and to evaluate the influence of different animal species on its proximate composition, physicochemical properties, and sensory attributes throughout the curing phase and in the finished product.

## Materials and methods

### Experimental design

A completely randomized design with a 3 × 2 factorial arrangement was used to compare the effect of species (cattle, buffalo, and camel) and curing period on the proximate composition, physicochemical characteristics, and sensory quality of air-dried cured meat “Grison”. Comparative analyses were conducted on meat samples every 3 days during a 12-day curing period at 4 °C, as well as in the corresponding Grison obtained after drying. A total of 162 samples from each species representing 2 topsides x 3 animals x 9 pieces x 3 replicates were divided into 6 groups, each have 3 groups and 3 subgroups.

### Raw materials

Nine each of 3-year-old uncastrated male cattle bulls (Native Seidi breed), water buffalo (*Bubalus bubalis*), and one-humped camel (*Camelus dromedaries*) with an average live body weight of 467.22 ± 10.84, 422.53 ± 4.94, and 368.23 ± 25.65 kg, respectively, were used in the study. Both cattle and buffalo bulls were farm-reared under veterinary supervision in Beni Sueif, Egypt. The animals were kept in separate pens on the same feed for 90 days before slaughter. The finishing ration was formulated of concentrates (14% crude protein), whole corn silage (33% DM), Alfa Alfa hay (89.8% DM), wheat straw (91% DM), limestone, sodium bicarbonate, salt, vitamins & minerals premixes, yeast, and other feed additives. While camels were reared in semi-arid pastures.

All animals were fasted for 12 h before slaughter, with free access to water. The animals were slaughtered according to traditional Halal protocol in Egypt’s main Cairo slaughterhouse. After carcass preparation, the hot carcass weight was recorded as 272.39 ± 4.94, 244.5 ± 4.23, and 209.75 ± 14.10 kg for cattle, buffalo, and camel, respectively. The topsides muscles from both sides of the three species were excised within three hours of slaughter, and transferred immediately in insulated cool boxes to the Faculty of Veterinary Medicine laboratory at Cairo University, where they were maintained in a chiller at 4 °C for 18 h before Grison processing. LobaChemie (Mumbai, India) supplied both sodium nitrite and potassium sorbate, while common salt and ground wild spices were purchased from local markets in Cairo, Egypt.

### Preparation of the raw materials

The two topside muscles from the three animals of each species in each replicate were entirely stripped of visible fat, connective tissue, and tendons. After trimming, the topsides of each species were dissected into separate cuts of about 1.75 kg each, randomly mixed, and divided into three groups with 9 pieces each. A curing mixture was prepared to provide 16.66% sodium chloride, 0.13% sodium nitrite, and 0.16% potassium sorbate (w/w meat). The spice blend was prepared by mixing 1 kg each of garlic powder and white pepper with 500 g each of cinnamon, cloves, and paprika. The spice mix was incorporated at a rate of 2 g/kg of meat.

### Processing of the Grison

After efficient trimming, the topside cuts were wrapped with the curing mixture and the spice blend. The meat was then stacked in a large container with bay leaf papers in between and cured for 12 days at 4 °C. The meat was manually rearranged upside down every 12 h, with proper mixing with the juices that came out of the meat. After the curing process, the meat pieces were carefully rinsed with running water and brushed to ensure that all of the curing mix and spices were removed. The cured meat pieces were pressed under a hydraulic pressing machine at 0.01 kg/m² for 2 h, then hung in a controlled air-conditioned room (18 °C, 60% RH, 2.7 m/s air flow) equipped with fans for two weeks. During storage, the meat was pressed once a week and dried for 4 h (35 °C, 30% RH, 2.7 m/s air flow) day after day, to lose about 35% of its original weight.

### Investigations

During the 12-day curing phase, meat samples were examined at 0 times, then every 3 days for proximate composition, pH value, thiobarbituric acid reactive substances (TBARS) content, salt concentration, and residual nitrite content. The finished product “Grison” was assayed for the same parameters in addition to the shear force, instrumental color attributes, and sensory characteristics. Each sample was analyzed three times, with three readings recorded for each parameter.

### Proximate composition

The moisture, protein, fat, and ash content were determined according to the AOAC^22^ methods. For moisture content, ten g samples were dried at 100 °C in a hot air oven (Precision™ Thermo 3510, Thermo Fisher Scientific, USA) until obtaining two successive constant weights. A 0.5 g sample was digested in concentrated H_2_SO_4_, distilled using the micro-Kjeldahl distillation unit (KT 200 Kjeltec™, FOSS, Denmark), and then the titratable HCL amount was multiplied by a 6.25 conversion factor to calculate the total protein percentage. The ether extractable fat was measured by drying a 10 g sample for 6–8 h at 125 °C, then extracting the fat using petroleum ether (40–60 °C) in a Soxhlet apparatus (LABTORN, LSOA-A12, UK). Finally, the ash percentage was determined by igniting a 5 g sample at 500–600 °C for 3 h in a muffle furnace (L 3/11, Nabertherm GmbH, Germany).

## Physicochemical analysis

### Determination of pH value

Five grams of each meat sample during the curing period and from the finished product were homogenized with 20 mL of distilled water for 15 s, following the procedure of Kandeepan et al.^23^. The pH value of the resulting slurry from each sample was measured three times using a pH meter (SensoDirect 110, Lovibond^®^, Germany) fitted with a probe-type electrode (SensoDirect 330, Lovibond^®^, Germany), and the average was computed.

### Thiobarbituric acid reactive substances (TBARS) content

Thiobarbituric acid reactive substances (TBARS) content was determined using the method described by Du and Ahn^24^ by homogenizing a 5 g meat sample with 15 mL deionized water. One mL of the homogenate was mixed with 50 µL of butylated hydroxytoluene (7.2 g/100 g) and 2 mL of a thiobarbituric acid/ trichloroacetic acid (TBA–TCA) solution (15 mM TBA, 15 g/100 g TCA). The mixture was mixed thoroughly, heated in a boiling water bath (Thermo Scientific SWB 15, Illinois, USA) for 15 min, cooled, centrifuged at 2500×g for 15 min using a laboratory centrifuge (CD-0412-50, Phoenix Instrument, Berlin, Germany), and the absorbance of the supernatant was measured at 531 nm using a spectrophotometer (S-1200 E, UNIO, SKU, USA). The TBARS content was reported as milligrams of malondialdehyde (MDA) per kilogram of sample.

### Salt concentration and residual nitrite content

Both salt concentration and residual nitrite content were determined following the method of AOAC^25^. The salt concentration was estimated by combining 1 g of the meat sample with 40 mL of silver nitrate (N/10) and 5 mL of nitric acid, then gently boiling for 15 min. After cooling, 50 mL of distilled water and 2 mL of saturated ferric ammonium sulfate solution were added, and the excess silver nitrate was titrated with N/10 ammonium thiocyanate solution using ferric ion as an indicator. The residual nitrite content was evaluated by heating 5 g of the sample with 300 mL of distilled water in a steam bath (Thermo Scientific SWB 15, Illinois, USA) at 80 °C for 2 h, then diluting to 500 mL and cooling before filtration. An aliquot (10 mL) of the filtrate was mixed with 2.5 mL sulfanilamide solution, allowed to stand for 5 min, and then combined with 2.5 mL N-(1-naphthyl) ethylenediamine (NED) reagent before being diluted to 50 mL. After 15 min, the absorbance of the developed color was measured at 540 nm against a reagent blank using a spectrophotometer (S-1200 E, UNIO, SKU, USA). The results were represented as a percentage of salt and ppm of nitrite.

### Shear force and color evaluation

For shear force investigation, finished product samples were cut into 2 cm thick steaks, and six cores (1.3 cm in diameter) were excised parallel to the muscle fiber orientation with a coring device. Each core was sheared once using a Warner-Bratzler shear force attachment on an Instron Universal Testing Machine (Model 2519 105, Instron Corp., Canton, MA, USA), which was outfitted with a 55-kg load cell and ran at a crosshead speed of 200 mm/min as the method described by Shackelford et al.^26^. Grison samples were cut into 30-mm slices and bloomed for 30 min at room temperature before being measured. Color parameters lightness (L*), redness (a*), and yellowness (b*) were measured using a calibrated colorimeter (CR-410, Konica Minolta, Japan) equipped with a D65 illuminant and a 10° viewing angle following the method described by Shin et al.^27^. The chroma (C*) (color saturation index) was calculated using the following formula: $C*=\sqrt{({a*)}^{2}+({b*)}^{2}}$ and the hue angle (*h°*) was calculated using the following formula: *H°* = arctan (b^*^/a^*^), and the ΔE values were calculated from the following equation, ${\Delta}\mathrm{E}=\sqrt[2]{{\left({\Delta}L*\right)}^{2}+{\left({\Delta}a*\right)}^{2}+{\left({\Delta}b*\right)}^{2}}$ where ΔL*, Δa*, and Δb* are the differences in lightness, redness, and yellowness (sample vs. fresh meat for each type of meat).

### Sensory analysis

A sensory laboratory with separate cabinets and standard facilities, including controlled lighting conditions and warm mineral water to clean the mouth between samples, was used for the sensory analysis. A 50-panel team (60% female and 40% male, ages 20–40) from the staff and students of the Department of Food Hygiene and Control, Faculty of Veterinary Medicine, Cairo University, Egypt, was carefully selected to participate in the panel analysis based on their previous experience in dry-cured meat products. The panel team also received three training sessions before the main sensory session to gain theoretical and practical experience with the sensory parameters of cured meat products. In addition, participants received comprehensive information about the complete plan of the study and the procedure used. The participation of the panel team was kept anonymous throughout the analysis, and they were free to leave the study at any moment^28^. The panelists assessed all samples with three batches each, including all trials spread consistently to cover three evaluation sessions in a single day. In each session, panelists received different batches of cattle, buffalo, and camel Grison in a randomized manner. Uniformly cut Grison slices from each batch were coded and presented to the panelists, who rated the color, flavor, juiciness, tenderness, and overall acceptability using a nine-point hedonic scale, where 1 represented extremely unacceptable, and 9 represented extremely acceptable^29^.

### Statistical analysis

The production of Grison from different animal species was replicated three times on different days. At each sampling time, three samples from each batch were analyzed three times for the different parameters, with three readings recorded to obtain 27 readings for each parameter. The proximate composition, pH value, TBARS content, salt concentration, and residual nitrite content were statistically analyzed during the curing phase and the finished product using a multiple linear model in SPSS 23.0 for Windows (SPSS Inc., Chicago, IL, USA). The meat type and curing times were used as the fixed factors, while the batch number was the random factor. For sensory analysis, the meat type was used as a fixed factor while the batch number, panel session, and panelists were the random factors. The one-way analysis of variance (ANOVA) was applied to compare the mean values of shear force and instrumental color parameters among the finished products prepared from the different types of meat. All values were presented as means ± standard error. Bonferroni’s multiple comparison test was used to identify significant differences at *P* < 0.05.

## Results

### Proximate composition

The results showed that both curing and drying reduced the moisture content while increasing the protein, fat, and ash content across different types of meat and product treatments (Table 1). During the curing phase, camel meat exhibited higher moisture and lower protein content than either cattle or buffalo meat (*P* < 0.05). Similar differences were observed in the final Grison product. Camel meat showed lower fat content from the 3rd day of curing until the end of the curing period compared with cattle meat (*P* < 0.05), whereas no difference was detected between camel and buffalo meat (*P* > 0.05). However, camel Grison recorded the lowest fat content compared to those processed from cattle and buffalo (*P* < 0.05). In contrast, camel meat presented the highest ash content, particularly on 0, 6th, and 12th days of curing, as well as in the final product, compared with cattle (*P* < 0.05), with no differences between camel and buffalo (*P* > 0.05). When comparing cattle and buffalo, cattle meat exhibited higher fat content on the 9th and 12th days of curing (*P* < 0.05), although no differences were observed in the final products (*P* > 0.05). Moreover, cattle meat showed higher protein content than buffalo meat, especially on the 3rd and 9th days of curing (*P* < 0.05); nevertheless, no difference was found between cattle and buffalo Grison (*P* > 0.05).

**Table 1:** Proximate composition (g/100 g) of different types of meat during the curing period and in the final Grison product.

	Salting time					Grison	*P* value
	0 time	3th day	6th day	9th day	12th day		Curing time
**Moisture**							
Cattle	75.45 ± 0.23^b, A^	61.22 ± 0.31^c, B^	60.41 ± 0.23^c, BC^	58.25 ± 0.63^b, C^	56.22 ± 0.29^c, D^	48.65 ± 0.34^c, E^	0.018
Buffalo	76.55 ± 0.21^ab, A^	63.90 ± 0.35^b, B^	62.54 ± 0.19^b, B^	60.23 ± 0.26^a, B^	58.02 ± 0.40^b, C^	49.35 ± 0.41^b, D^	0.021
Camel	78.0 ± 0.65^a, A^	65.65 ± 0.35^a, B^	63.53 ± 0.26^a, B^	61.60 ± 0.77^a, B^	59.45 ± 0.32^a, C^	52.81 ± 0.25^a, D^	0.029
*P* value type of meat	0.003	0.012	0.034	0.022	0.009	0.018	
**Protein**							
Cattle	20.84 ± 0.28^a, C^	23.32 ± 0.13^a, C^	24.23 ± 1.01^a, C^	25.40 ± 0.45^a, C^	26.45 ± 0.11^a, B^	30.96 ± 0.37^a, A^	0.033
Buffalo	21.10 ± 0.09^a, E^	22.64 ± 0.07^b, D^	22.90 ± 0.37^a, CD^	23.71 ± 0.23^b, BC^	24.21 ± 0.52^a, B^	31.12 ± 0.30^a, A^	0.037
Camel	19.63 ± 0.45^b, D^	20.09 ± 0.46^c, CD^	20.92 ± 0.12^a, BC^	21.15 ± 0.26^c, BC^	21.91 ± 0.38^b, B^	27.73 ± 0.65^b, A^	0.019
*P* value type of meat	0.034	0.027	0.018	0.006	0.011	0.023	
**Fat**							
Cattle	1.83 ± 0.22^a, D^	2.84 ± 0.08^a, D^	3.14 ± 0.48^a, C^	3.85 ± 0.15^a, BC^	4.23 ± 0.13^a, B^	5.19 ± 0.09^a, A^	0.030
Buffalo	1.43 ± 0.17^a, D^	1.72 ± 0.29^ab, CD^	1.72 ± 0.39^ab, CD^	2.24 ± 0.1^b, C^	3.14 ± 0.07^b, B^	4.92 ± 0.07^a, A^	0.037
Camel	0.54 ± 0.02^b, D^	1.25 ± 0.11^b, CD^	1.33 ± 0.36^b, CD^	1.92 ± 0.19^b, C^	2.94 ± 0.41^b, B^	4.02 ± 0.08^b, A^	0.011
*P* value type of meat	0.011	0.021	0.031	0.025	0.019	0.024	
**Ash**							
Cattle	0.76 ± 0.09^b, C^	11.23 ± 0.26^a, B^	12.18 ± 0.18^b, B^	12.46 ± 1.17^a, AB^	12.58 ± 0.79^b, B^	14.16 ± 0.38^b, A^	0.020
Buffalo	0.89 ± 0.03^ab, E^	11.59 ± 0.48^a, D^	12.68 ± 0.22^ab, C^	13.22 ± 0.13^a, C^	14.03 ± 0.27^a, B^	14.44 ± 0.21^ab, A^	0.017
Camel	1.10 ± 0.04^a, D^	12.02 ± 0.44^a, C^	13.49 ± 0.29^a, BC^	14.66 ± 0.95^a, AB^	14.74 ± 0.47^a, AB^	15.31 ± 0.26^a, A^	0.026
*P value* type of meat	0.022	0.028	0.014	0.049	0.027	0.042	

^a−c^ Means with different superscripts in the same column differ significantly at *P* < 0.05.

^A−D^ Means with different superscripts in the same row differ significantly at *P* < 0.05.

### pH value, TBARS content, salt concentration, and residual nitrite content

The results indicated that pH values increased during the curing period up to day 6 and subsequently decreased during the remaining curing period and drying process in meat from different species of animals and their final products (*P* < 0.05). Camel meat and its corresponding Grison exhibited the highest pH values, whereas the cattle counterparts showed the lowest values (*P* < 0.05). Furthermore, the curing and drying processes increased the TBARS content in all samples. Camel meat exhibited lower TBARS content than cattle and buffalo meat up to the 6th day of curing (*P* < 0.05). However, on the 9th and 12th day, no changes were observed between all types of meat (*P* > 0.05). In the finished product, cattle Grison had the highest value (*P* < 0.05), with no differences observed among buffalo and camel products (*P* > 0.05). Both curing and drying increased the salt content while lowering the residual nitrite content. During the curing period, cattle meat exhibited lower salt levels than buffalo and camel meat, especially during the 3rd to 9th days (*P* < 0.05). However, on the 12th day, the highest salt content was found in camel meat (*P* < 0.05), with no difference between cattle and buffalo meat (*P* > 0.05). In the final Grison, cattle had the lowest salt concentration, while camel meat had the highest values (*P* < 0.05). Using different types of meat during Grison processing led to substantial differences in residual nitrite content, with camel meat having the highest value and cattle having the lowest content up to the 9th day of curing (*P* < 0.05). However, by the end of the curing period and in the finished Grison products, no differences were observed between cattle and buffalo (*P* > 0.05) (Table 2).

**Table 2:** pH value, TBARS content, salt concentration, residual nitrite content of different types of meat during the curing period, and in the final Grison product.

	Salting time					Grison	*P* value
	0 time	3th day	6th day	9th day	12th day		Curing time
**pH value**							
Cattle	5.63 ± 0.12^b, B^	5.77 ± 0.21^b, A^	5.80 ± 0.11^c, A^	5.60 ± 0.17^b, B^	5.55 ± 0.23^b, C^	5.50 ± 0.14^c, C^	0.001
Buffalo	5.65 ± 0.09^b, D^	5.80 ± 0.05^b, B^	5.93 ± 0.14^b, A^	5.84 ± 0.13^a, B^	5.72 ± 0.019^a, C^	5.65 ± 0.23^b, D^	0.021
Camel	5.88 ± 0.16^a, C^	5.94 ± 0.11^a, B^	6.00 ± 0.06^a, A^	5.86 ± 0.22^a, C^	5.80 ± 0.08^a, E^	5.75 ± 0.16^a, D^	0.022
*P*-value type of meat	0.040	0.061	0.052	0.34	0.29	0.031	
**TBARS (mg mal./kg)**							
Cattle	0.08 ± 0.008^a, C^	0.11 ± 0.005^a, C^	0.19 ± 0.014^a, BC^	0.27 ± 0.03^a, B^	0.29 ± 0.09^a, B^	0.46 ± 0.02^a, A^	0.002
Buffalo	0.07 ± 0.008^a, D^	0.11 ± 0.015^a, CD^	0.18 ± 0.018^a, C^	0.21 ± 0.07^a, BC^	0.28 ± 0.02^a, B^	0.39 ± 0.01^b, A^	0.015
Camel	0.02 ± 0.003^b, C^	0.05 ± 0.008^b, C^	0.09 ± 0.008^b, BC^	0.18 ± 0.08^a, AB^	0.28 ± 0.02^a, A^	0.30 ± 0.02^b, A^	0.011
*P*-value type of meat	0.019	0.21	0.003	0.001	0.024	0.028	
**Salt (g/100 g)**							
Cattle	1.52 ± 0.15^a, D^	7.05 ± 0.05^b, C^	7.34 ± 0.03^b, BC^	7.59 ± 0.08^b, B^	7.81 ± 0.02^b, B^	8.01 ± 0.01^b, A^	0.023
Buffalo	1.72 ± 0.11^a, B^	7.81 ± 0.06^a, A^	7.95 ± 0.03^a, A^	7.95 ± 0.03^a, A^	7.97 ± 0.01^b, A^	8.05 ± 0.01^ab, A^	0.012
Camel	2.04 ± 0.14^a, B^	7.91 ± 0.03^a, A^	7.96 ± 0.06^a, A^	7.97 ± 0.02^a, A^	7.98 ± 0.01^a, A^	8.07 ± 0.01^a, A^	0.015
*P*-value type of meat	0.018	0.022	0.013	0.031	0.037	0.026	
**Residual nitrite (ppm)**							
Cattle	118.33 ± 6.13^a, B^	285.66 ± 7.22^c, A^	263.33 ± 3.38^c, C^	226.37 ± 7.50^c, D^	106.21 ± 2.60^b, E^	103.50 ± 2.11^b, D^	0.029
Buffalo	119.66 ± 4.91^a, A^	313.00 ± 3.78^b, A^	290.33 ± 2.60^b, B^	259.77 ± 7.90^b, C^	113.34 ± 5.10^ab, D^	104.43 ± 2.40^b, E^	0.021
Camel	122.33 ± 5.23^a, B^	358.67 ± 9.40^a, A^	324.00 ± 3.46^a, C^	295.00 ± 6.00^a, D^	121.93 ± 2.00^a, E^	115.95 ± 1.61^a, E^	0.013
*P*-value type of meat	0.015	0.017	0.022	0.031	0.045	0.039	

^a−c^ Means with different superscripts in the same column differ significantly at *P* < 0.05.

^A−D^Means with different superscripts in the same row differ significantly at *P* < 0.05.

### Shear force, color evaluation, and sensory analysis

Processing Grison with different types of meat resulted in significant changes in both shear force values and product color indices. Buffalo Grison showed the highest shear force value, followed by camel and cattle (*P* < 0.05). The Grison prepared from cattle meat exhibited higher (L*) and (b*) values compared with buffalo and camel (*P* < 0.05). In contrast, camel Grison showed the highest (a*) value (*P* < 0.05), while no differences were observed between camel and buffalo Grison in (L*) and (b*) values (*P* > 0.05). The derived color indices followed a similar pattern. Chroma (c*) values were higher in camel Grison and lower in cattle Grison, whereas (*h°*) values showed an opposite pattern (*P* < 0.05). Moreover, ΔE values were the lowest in cattle Grison (*P* < 0.05), with no differences observed between buffalo and camel products (*P* > 0.05). Sensory panel scores of various trials revealed that camel Grison had a considerably higher color score than those made from cattle or buffalo (*P* < 0.05). Furthermore, camel Grison had the highest flavor score, while buffalo had the lowest score (*P* < 0.05). In addition, buffalo Grison had the lowest juiciness, tenderness, and overall acceptability scores (*P* < 0.05), with no differences found between cattle and camel products (*P* > 0.05) (Table 3).

**Table 3:** Shear force, instrumental color, and sensory profile of the final Grison product.

	Cattle	Buffalo	Camel	*P* value
Shear force (Newtons)	49.55 ± 1.34^c^	84.20 ± 1.93^a^	68.66 ± 5.00^b^	0.033
**Instrumental color**				
Lightness (L*)	39.93 ± 0.44^a^	36.11 ± 0.77^b^	35.89 ± 1.2^b^	0.048
Redness (a*)	8.15 ± 0.17^c^	9.54 ± 0.19^b^	11.00 ± 0.3^a^	0.028
Yellowness (b*)	2.67 ± 0.33^a^	1.09 ± 0.08^b^	0.58 ± 0.19^b^	0.036
Chroma (c*)	8.58 ± 0.25^c^	9.67 ± 0.27^b^	11.02 ± 0.29^a^	0.012
Hue (*h*°)	18.14 ± 0.19^a^	6.52 ± 0.34^b^	3.02 ± 0.17^c^	0.018
ΔE	1.95 ± 0.20^b^	2.16 ± 0.25^a^	2.37 ± 0.30^a^	0.020
**Sensory analysis**				
Color	7.35 ± 0.34^b^	6.33 ± 0.5^c^	8.78 ± 0.11^a^	0.028
Flavor	7.51 ± 0.42^ab^	6.49 ± 0.56^b^	8.20 ± 0.41^a^	0.009
Juiciness	8.33 ± 0.26^a^	6.11 ± 0.29^b^	8.04 ± 0.19^a^	0.001
Tenderness	8.35 ± 0.14^a^	6.39 ± 0.53^b^	8.06 ± 0.10^a^	0.037
Overall acceptability	7.93 ± 0.05^a^	6.33 ± 0.43^b^	8.27 ± 0.06^a^	0.019

^a−c^Means with different superscripts in the same row differ significantly at *P* < 0.05.

## Discussion

In general, both curing and drying have a substantial impact on the proximate composition of meat during the curing period as well as the finished products. Curing induced a noteworthy decline in moisture content due to water loss while salt penetrates the meat matrix, resulting in a relative increase in protein, fat, and ash concentrations in the salted meat due to the concentration of nutrients rather than the building up of new components. Such observation was in agreement with the finding of Rahmani et al.^30^. The variations in the proximate composition between the different Grison treatments can be primarily attributed to the inherent variances in the proximate composition of the meat from different species of animals used in their formulations. Previous studies have reported that camel meat generally contains higher levels of moisture and ash but lower levels of protein and fat than bovine meat^31^. Kadim et al.^32^. attributed the higher moisture content of camel meat to its high potassium content, which regulates body fluid as a physiological adaptation to arid environments. Additionally, the increased ash content in camel meat may reflect its higher mineral content and lower intramuscular fat level compared with cattle. Suliman and Fadlalmola^33^ reported that lower fat concentrations allow a higher relative proportion of protein and minerals (ash) per gram of meat. In contrast, cattle’s increased fat content is related to lower moisture content, which is consistent with Watanabe et al.^34^. From this perspective, the substantially higher protein content in buffalo Grison compared with that produced from camel meat can be attributed to the intrinsic characteristics of buffalo meat, which is known to possess higher protein levels and lower fat deposition than meat from other species^35^.

In general, the pH value, TBARS content, salt concentration, and residual nitrite content were significantly impacted during both the curing and drying phases of Grison production. The increase in pH value during the first 6 days of the curing period may be attributed to the proteolytic activity of microorganisms. Virgili et al.^36^ established that a low level of common salt in cured products generates alkaline compounds due to microbial proliferation, which subsequently increases the pH value. Moreover, Garcia-Rey et al.^37^ also correlated low salt content of dry-cured meat products with protein hydrolysis and possible harmful effect to both texture and taste. In contrast, the decrease in pH value observed at the end of the curing period and in the final products can be attributed to the elevated salt content. High salt content is connected with the reduction in water activity, inhibition of microbial and enzymatic activity, and reduction in the formation of alkaline metabolites^38^. Additionally, the increase in lactic acid concentration due to the growth of lactic acid bacteria under high-salt conditions may further contribute to the observed decline in pH values as reported by Fraqueza et al.^39^. The inherently lower glycogen content and higher initial pH value of camel meat^40^ may explain the higher pH values during the curing process and its corresponding Grison products.

The TBARS contents increased during the curing period across all types of meat and throughout the drying process in the various Grison products. Lorenzo^41^ indicated that the increase in malondialdehyde content is associated with oxidative reactions that may be intensified by the pro-oxidant effects of salt and metallic ion impurities present in curing salt. The lower TBARS levels in camel Grison could be attributed to several mechanisms, including the significantly lower fat content and higher residual nitrite recorded in the present study, as well as the elevated glutathione oxidase and catalase activities, as established by Gheisari et al.^42^. Although camel meat has low intramuscular fat, it is more liable to lipid oxidation due to its higher percentage of unsaturated fatty acids^13^, a matter which substantiates the role of other mechanisms in lipid stabilization of dry-cured meat products. The high bioactive compounds, e.g., glutathione peroxidase and catalase in camel meat, may contribute to the enzymatic antioxidant defense system and limit oxidative deterioration^43^. Moreover, the higher residual nitrite in camel compared to cattle and buffalo Grison (Table 2) may be associated with reduced TBARS readings due to the powerful antioxidant effect of nitrite^44^. The reduced TBARS readings seen in buffalo Grison compared to cattle-based formulations may be attributed to its lower lipid content (Table 2). Failla et al.^45^ linked both low intermolecular fat content and high oleic acid level with significant antioxidant activity in buffalo meat.

The meat from different species of animals has an important impact on modulating salt diffusion and residual nitrite content throughout the curing process. Furthermore, both the curing time and drying processes considerably add another effect. Camel meat has higher salt content and residual nitrite levels than either cattle or buffalo, which is most likely due to the greater diffusion of curing salts into camel meat owing to its higher moisture and lower fat contents. Sheng et al.^46^ found that the lipid composition and muscle structure play critical roles in controlling salt diffusion, and Lebert and Daudin^47^ observed that muscles with higher fat levels have greater resistance to the diffusion of aqueous solutes such as sodium chloride. The reduction in residual nitrite content during curing and drying may also be correlated with the conversion of nitrite to nitric oxide, which subsequently combines with myoglobin to develop the characteristic red color of cured meat^48^.

The discrepancies in shear force values among various Grison treatments may be attributed to variations in protein, fat, connective tissue content, and the coarseness of muscle fibers among different types of meat. The results of shear force revealed that buffalo Grison had the highest shear, followed by camel, and finally, cattle meat. Nakamura et al.^49^. reported a negative correlation between shear force and lipid content, and a positive correlation with connective tissue content of meat. In this respect, the higher shear force reported in buffalo Grison may be attributed to its higher myofibrillar protein, lower fat content, thicker muscle fiber diameter, and shorter sarcomere length than cattle, as previously reported by Shahein et al.^50^. Furthermore, Eskandari et al.^18^. stated that camel meat contains lower fat and higher connective tissue levels than cattle meat, which may be the main reasons for the higher shear force values observed in camel Grison.

Instrumental color evaluation of Grison prepared from meat from different species of animals revealed notable variations in both primary (L*, a*, and b*) and derived (c*, *h°*, and ΔE) color parameters. Cattle Grison exhibited higher (L*), (b*), and (*h°*) values but a lower (c*) value compared with buffalo and camel products. The relatively higher (L*) value in cattle Grison may be attributed to its lower moisture and higher fat content, which is consistent with Young et al.^51^., who reported an inverse relationship between water content and food lightness. In contrast, camel Grison showed the highest (a*) and (c*) values and the lowest (*h°*) value, indicating a shift toward redder tones. The higher residual nitrite and moisture contents, together with the lower fat content of camel Grison observed in this study, may explain the enhanced redness of camel products as described by Posthuma et al.^52^. Moreover, Wali et al.^40^ attributed the red color of camel meat to elevated hemoglobin and myoglobin levels. These instrumental findings were consistent with sensory evaluations, where camel Grison received the highest color scores, likely due to its uniform and visually appealing red hue compared with the more intense but less uniform color of cattle and buffalo Grison.

The relatively higher (b*) value in cattle Grison may also reflect its lower antioxidant activity compared with camel and buffalo products. Previous studies reported higher glutathione peroxidase and catalase activities in camel meat^43^ and higher oleic acid content in buffalo meat^45^, both of which may contribute to improved oxidative stability. In addition, the higher residual nitrite content observed in camel and buffalo Grison may represent another factor contributing to its lower (b*) value compared with cattle counterparts, as nitrite exhibits potent antioxidant activity by inhibiting myoglobin oxidation and limiting (b*) development^44^. The ΔE values, representing total color difference, were the lowest in cattle Grison, suggesting greater color stability during processing, whereas higher ΔE values in camel and buffalo products indicate more pronounced color changes during curing and drying. This observation may be explained based on the finding that cattle meat has low myoglobin, while both buffalo and camel have higher myoglobin concentration and iron levels^14,53^. Overall, the observed trends in instrumental color parameters aligned well with sensory assessments, confirming that camel Grison exhibited the most visually appealing color among the tested meat types.

Camel Grison likewise received the highest flavor ratings, presumably due to its higher salt content, which contributes to the distinctive flavor of dry-cured meat products. In contrast, buffalo Grison received poorer flavor, juiciness, tenderness, and overall acceptability scores, which could be attributed to its higher myoglobin concentration than other animal species. Naveena et al.^54^ observed that both increased heme iron concentration and myoglobin content increase the meat hardness. The relationship between myoglobin/heme iron content and meat tenderness is not considered a direct causal effect. The higher myoglobin levels are characteristic of muscles that possess greater connective tissue content and collagen crosslinking. These structural properties are well known to increase shear force and reduce tenderness^55^. The increased juiciness and tenderness of cattle and camel Grison could be attributed to the higher intramuscular fat content in cattle and the higher moisture level in camel flesh. This explanation aligns with Warner^56^, who reported a positive relationship between tenderness and juiciness and the intramuscular fat and moisture content of meat.

Although the present study provides valuable information on the proximate composition, physicochemical, and sensory characteristics of Grison prepared from cattle, buffalo, and camel meat during processing and in the final product, several limitations should be acknowledged. Microbiological quality was not evaluated, which could provide additional insight into product safety and shelf life stability. In addition, the fatty acid profile was not determined, which may be important for understanding the nutritional and functional properties of this product. Furthermore, the study did not include a storage period evaluation, which could help assess changes in quality parameters over time. Future studies are recommended to investigate the microbiological quality and safety of Grison products, as well as to evaluate their fatty acid profile. Moreover, further research should consider monitoring the quality changes during the storage period to better understand product stability and shelf-life behavior.

## Conclusion

The meat from different animal species influences the proximate composition, physicochemical characteristics, and sensory quality of Grison. Grison made from camel meat was comparable to that produced from cattle, with higher color scores, whereas buffalo Grison exhibited lower tenderness, as indicated by its higher shear force value. These results provide useful insights into the potential effect of meat type on Grison quality. However, future work should focus on improving the sensory quality of buffalo Grison to enhance its tenderness and juiciness, with the aim of increasing its comparability to traditional cattle Grison product. In addition, further investigations are required to evaluate the microbiological quality and safety of Grison products. The assessment of fatty acid profiles, as well as changes during storage, may also provide a more comprehensive understanding of product quality and stability.

## Data Availability

Data will be made available on request.
